# The Retreat from Locative Overgeneralisation Errors: A Novel Verb Grammaticality Judgment Study

**DOI:** 10.1371/journal.pone.0097634

**Published:** 2014-05-15

**Authors:** Amy Bidgood, Ben Ambridge, Julian M. Pine, Caroline F. Rowland

**Affiliations:** Department of Psychological Sciences, University of Liverpool, Liverpool, Merseyside, United Kingdom; Stony Brook University, United States of America

## Abstract

Whilst some locative verbs alternate between the ground- and figure-locative constructions (e.g. *Lisa sprayed the flowers with water/Lisa sprayed water onto the flowers*), others are restricted to one construction or the other (e.g. *Lisa filled water into the cup/*Lisa poured the cup with water). The present study investigated two proposals for how learners (aged 5–6, 9–10 and adults) acquire this restriction, using a novel-verb-learning grammaticality-judgment paradigm. In support of the semantic verb class hypothesis, participants in all age groups used the semantic properties of novel verbs to determine the locative constructions (ground/figure/both) in which they could and could not appear. In support of the frequency hypothesis, participants' tolerance of overgeneralisation errors decreased with each increasing level of verb frequency (novel/low/high). These results underline the need to develop an integrated account of the roles of semantics and frequency in the retreat from argument structure overgeneralisation.

## Introduction

As adults, we have the capacity for enormous creativity in language production: we often produce utterances that we have never heard. To reach this stage, children must acquire the grammar of the ambient language by forming generalisations about that language from the input. However, children must also learn to restrict these generalisations in order to avoid producing ungrammatical utterances (e.g. *I don't want it because I spilled it of orange juice [ = I spilled orange juice onto my toast], [1]).

Pinker [2] listed various grammatical constructions that have two alternating forms. The locative construction, for example, alternates between the ground- (or container-) locative, as in *The farmer loaded the wagon with hay*, and the figure- (or contents-) locative, as in *The farmer loaded hay into the wagon*. In the first sentence, the wagon is most affected, as it changes state from empty to full. In the second sentence, it is the hay that is most affected, as it is moved to a specific location; the wagon may or may not end up full. Pinker ([2], page 79) described this change in how the event is construed as a “gestalt shift”. (For earlier work on these constructions, see e.g. [3]–[7]).

When children hear verbs used in both the ground- and figure-locative constructions (*load*, *spray*, *stuff*, etc.), they may create a generalisation that any verb used in one of these constructions can also be used in the other, and this works well for some verbs. A child hearing *You splashed me with water*, a ground-locative construction, might generalise to the figure-locative construction to produce the grammatical utterance, *You splashed water onto me*. However, some English verbs, such as *fill* and *cover*, can only be used in the ground-locative construction (ground-only verbs) and generalising these verbs to the figure-locative construction would produce an ungrammatical utterance, such as *We filled toys into the box. Conversely, some verbs, such as *pour* and *spill*, can only be used in the figure-locative construction (figure-only verbs). Generalising these verbs to the ground-locative construction would similarly produce overgeneralisation errors, such as *Daddy poured my cup with juice.

One factor that could contribute to the retreat from overgeneralisation errors is parental feedback: so-called ‘negative evidence’. It is undoubtedly the case that some parents provide feedback on errors that their children make, either through direct correction (e.g. C: *I filled mud into the hole, M: *No, say “I filled the hole with mud”*) or implicitly, via rephrasing (e.g. M: *That's right, you filled the hole with mud*), facial expressions, misunderstandings or requests for clarification. Whilst evidence suggests that such feedback is helpful [8], children are unlikely to receive sufficient feedback of this type to account entirely for their retreat from overgeneralisation errors, particularly for low frequency verbs. Furthermore, some examples of parent-child interactions suggest that such feedback may have only a limited effect on children's language production (for reviews, see e.g. [2], pages 9–14; [9]).

The current paper investigates the extent to which two mechanisms constitute a solution to the ‘no negative evidence’ problem [10] and therefore explain the retreat from overgeneralisation with locative constructions. The first of these is Pinker's [2] semantic verb class hypothesis: while evidence exists in support of this account, previous studies have primarily focussed on errors involving the transitive-causative and dative constructions, which, for reasons outlined in the following section, do not constitute as strong a test of the hypothesis. The second mechanism is statistical learning, in the form of entrenchment [11] or preemption [12]. Again, the locative alternation is a particularly good test of these hypotheses, as detailed below.

### The semantic verb class hypothesis

Pinker's [2] *semantic verb class hypothesis* attempts to explain how children's developing knowledge of verb semantics could explain the retreat from overgeneralisation errors. The proposed mechanism involves innate linking rules, which link generic semantic structures (‘thematic cores’) to verb argument structures: all verbs with the same thematic core are licensed in the same argument structure. These groups of verbs are known as *broad semantic classes*.

Some verbs, such as *spray* and *load*, can appear in more than one argument structure. Once children hear such examples, *broad-range rules* are formed (although the set of possible alternations is constrained by the innate linking rules). These allow verbs in related broad classes, such as figure and ground locative verbs, to alternate between the two structures. Until this point in development, learning is conservative and production is restricted to the use of verbs only in argument structures already heard by the child.

Of course, not all verbs that are grammatical in one locative construction are grammatical in the other, and this is due to idiosyncratic differences between verbs. Pinker ([2], pages 273–4) proposed that, by replacing “each idiosyncratic piece of information with a parameter” and matching verbs on this more detailed level of semantics, *narrow semantic classes* (or ‘subclasses’) are formed. It is only membership in an alternating narrow class that enables a verb to be used grammatically in the other argument structure, via a *narrow-range rule*.

According to the semantic verb class hypothesis, the cause of children's overgeneralisation errors is that children do not initially have well-developed knowledge of verb semantics and do not necessarily know enough verbs in each narrow class for these classes to have been accurately formed. Thus, overgeneralisations occur as children occasionally apply the broad-range rule to some verbs to which a narrow-range rule would not apply. There is some evidence that children know that these productive forms are ungrammatical ([2], page 322–4). Children retreat from error as the operation of narrow-range rules gradually supersedes that of broad-range rules; the broad-range rules do remain in place, however, and enable adults to produce ‘Haigspeak’ utterances (which the speaker/writer again does not necessarily consider to be grammatical, [2], pages 152–160).

Pinker ([2], pages 126–127) specifies 15 narrow subclasses for locatives and allocates each of the 146 verbs to one of these subclasses (with two exceptions, *wrap* and *string*, which may each be the only members of their own respective subclasses). The defining semantics of each subclass specify whether the verbs contained within it can alternate between constructions, via a narrow-range rule, although even alternating classes have a bias towards one of the two constructions. Table 1 (adapted from [13], page 262, based on [2], pages 126–127) details the 15 subclasses.

**Table 1:** Pinker's (1989) narrow-range subclasses for locative verbs, adapted from Ambridge, Pine and Rowland (2012).

**Figure- (content-) oriented (** ***into/onto*** ** verbs)**	***Smear*** **-type, Alternating (** ***N*** ** = 10), designated reference category.** Simultaneous forceful contact and motion of a mass against a surface	*brush, dab, daub, plaster, rub, slather, smear, smudge, spread, streak*
	***Stack*** **-type, Alternating (** ***N*** ** = 3).** Vertical arrangement on a horizontal surface	*heap, pile, stack*
	***Spray*** **-type, Alternating (** ***N*** ** = 7).** Force is imparted to a mass, causing ballistic motion in a specified spatial direction along a trajectory	*inject, spatter, splash, splatter, spray, sprinkle, squirt*
	***Scatter*** **-type, Alternating (** ***N*** ** = 4).** Mass is caused to move in a widespread or nondirected distribution	*bestrew, scatter, sow, strew*
	***Pour*** **-type, Content-only (** ***N*** ** = 10).** A mass is enabled to move via the force of gravity	*dribble, drip, drizzle, dump, ladle, pour, shake, slop, slosh, spill*
	***Coil*** **-type, Content-only (** ***N*** ** = 6).** Flexible object extended in one dimension is put around another object (preposition is *around*)	*coil, spin, twirl, twist, whirl, wind*
	***Spew*** **-type, Content-only (** ***N*** ** = 8).** Mass is expelled from inside an entity	*emit, excrete, expectorate, expel, exude, secrete, spew, vomit*
	***Glue*** **-type, Content-only (** ***N*** ** = 9).** Verbs of attachment	*attach, fasten, glue, nail, paste, pin, staple, stick, tape*
**Ground- (container-) oriented (** ***with*** ** verbs)**	***Stuff-*** **type, Alternating (** ***N*** ** = 6).** A mass is forced into a container against the limits of its capacity	*cram, crowd, jam, pack, stuff, wad*
	***Load*** **-type, Alternating (** ***N*** ** = 3).** A mass of a size, shape, or type defined by the intended use of a container is put into the container, enabling it to accomplish its function	*load, pack, stock*
	***Fill*** **-type, Container-only (** ***N*** ** = 21).** A layer completely covers a surface	*bandage, blanket, coat, cover, deluge, douse, edge, encrust, face, fill, flood, inlay, inundate, line, occupy, pad, pave, plate, shroud, smother, tile*
	***Pollute*** **-type, Container-only (** ***N*** ** = 22).** Addition of an object or mass to a location causes an aesthetic or qualitative, often evaluative, change in the location	*adorn, burden, clutter, deck, dirty, embellish, emblazon, endow, enrich, festoon, garnish, imbue, infect, litter, ornament, pollute, replenish, season, soil, stain, tint, trim*
	***Soak*** **-type, Container-only (** ***N*** ** = 15).** A mass is caused to be coextensive with a solid or layer-like medium	*drench, impregnate, infuse, interlace, interlard, interleave, intersperse, interweave, lard, ripple, saturate, soak, stain, suffuse, vein*
	***Clog*** **-type, Container-only (** ***N*** ** = 12).** An object or mass impedes the free movement of, from, or through the object in which it is put	*block, choke, clog, dam, plug, stop up, bind, chain, entangle, lash, lasso, rope*
	***Bombard*** **-type, Container-only (** ***N*** ** = 8).** A set of objects is distributed over a surface	*bombard, blot, dapple, riddle, speckle, splotch, spot, stud*
**Alternating verbs with “unique geometry” that do not fit into the above classes (** ***N*** ** = 2)**	Static of a linear object along a surface	*string*
	A flexible object conforms to part of the shape of an object along two or more orthogonal dimensions	*wrap*

Further work has since been conducted aimed at defining the nature of the verb classes more precisely (e.g. [14], [15]). However, this work does not changes the basic prediction of the semantic verb class hypothesis to be tested here, that children's production of, and retreat from, overgeneralisation errors will be predicted by their knowledge of the semantic class of the verb. In the present study, all of the verbs chosen were classified in the same way by both Pinker [2] and Levin [15], although it is worth noting that the organisation of verbs into classes of this kind is not universally accepted (e.g. [10]–[12], [16], [17]). It is also worth noting that the semantic verb class hypothesis cannot explain verb frequency effects, which are also pervasive in the literature (as reviewed below). Indeed, some authors (e.g. [18]) have argued that apparent semantic verb class effects are epiphenomenal, with learners acquiring verbs' argument structure restrictions solely on the basis of surface-based statistical learning mechanisms such as entrenchment and preemption. It is to these mechanisms that we now turn.

### The frequency hypothesis

Various accounts have attempted to explain how children are able to learn which verbs can be used in which constructions based on statistical properties of the input (e.g. [19], [20]). For example, the entrenchment hypothesis (e.g. [11], [21], [22]) proposes that, although children may be aware that it is possible to use certain verbs in two alternating constructions, such as the ground- and figure-locative constructions, they gradually learn that this is not the case for all verbs. While children hear figure-only verbs, such as *pour*, frequently in their input, they never hear them in the ground-locative construction. Eventually, this leads children to infer that, if it were possible to use *pour* in this construction, they “would have heard it by now”, and hence that ground-locative uses of this verb are ungrammatical for adult speakers. An account that includes a related statistical mechanism (alongside a semantic element) is preemption (e.g. [12], [23]–[25]). This account proposes that only uses of the verb in a different grammatical pattern that nevertheless yields the same meaning will lead to the inference that the non-attested form is ungrammatical. For example, utterances such as *She poured water into the cup* would pre-empt *She poured the cup with water, but other semantically more distant uses (e.g., *It's pouring with rain*) would not (or, at least, would do so to a lesser degree).

Ambridge, Pine and Rowland [13] attempted to distinguish between the effects of entrenchment and preemption on the retreat from overgeneralisation in the locative construction, suggesting that both may play a role. However, their entrenchment and preemption predictors were highly correlated, which made it difficult to distinguish effects of one from the other (see also [26]). For this reason, differentiating between entrenchment and preemption is beyond the scope of the present study (see also e.g. [27], page 2; [28], page 612). For the remainder of this paper, we will therefore simply refer to the ‘frequency hypothesis’. Our findings and conclusions could apply equally to the entrenchment and preemption hypotheses.

### Existing evidence for the two accounts

Previous studies have provided evidence in support of both the semantic verb class hypothesis and statistical learning accounts. However, these have primarily been restricted to overgeneralisation errors relating to the causative alternation, such as *Homer broke the plate*/*The plate broke* (e.g. [26], [29]–[35]). While these studies provide some support for both the semantic verb class hypothesis and the frequency hypothesis, any successful account must be able to deal with all of the alternations for which overgeneralisation errors are sometimes observed. Ambridge, Pine, Rowland and Chang [36] tested the predictions of the semantic verb class and entrenchment hypotheses with the dative construction, finding support for both theories, but only in their adult participants (see also [37] for support for broad and narrow verb classes in the dative construction).

So, while the results of studies involving the causative alternation appear to be consistent with both the semantic verb class and frequency hypotheses, both seem to struggle in the domain of the dative alternation. One possible explanation is that the dative is a special case, and that the semantic verb class and frequency hypotheses can explain the retreat from overgeneralisation across a range of different constructions. Another is that it is the causative alternation that is the special case, with other constructions showing no semantic class and frequency effects. The aim of the present paper is, thus, to test the scope of the two hypotheses by testing their predictions against a third alternation: the locative.

### The locative alternation

Like the dative, the locative alternation contains two relatively low frequency constructions with fine-grained distinctions between the relevant narrow semantic subclasses, and therefore constitutes a particularly good test case for both hypotheses. It provides a strong test of the semantic verb class hypothesis because of the sometimes very subtle differences between the narrow subclasses (see Table 1). For example, with alternating *spray*-type verbs, a mass is *caused* to move via a force imparted upon it whereas, with ground-only *pour*-type verbs, a mass is simply *enabled* to move via the force of gravity. In contrast, differences between subclasses for the causative alternation seem more clear-cut: For example, verbs specifying the *manner* of motion, such as *bounce* (*The ball bounced/Bart bounced the ball*), alternate whereas verbs that specify the *direction* of motion, such as *fall* (The ball fell/*Bart fell the ball), do not ([2], pages 130–4). In addition to the subtle subclass distinctions in the locative alternation, for children to form the appropriate subclasses, they would need to be able to observe the differences between them. Again, this seems far less plausible for locative verbs than for causative verbs since, in the locative example above, both the forces involved (e.g. gravity) and the subtle difference between causing and enabling motion are difficult to observe.

Like the dative, the locative alternation also provides a strong test of the frequency hypothesis due to the relatively low frequency of locative verbs, particularly in comparison with verbs involved in the causative alternation. A paucity of locative verbs (and, presumably, constructions) in the input could make it difficult for statistical learning mechanisms to operate.

A further advantage of studying the locative construction, in this case over both the causative and the dative constructions, is that it appears to be truly productive in both directions. With regard to the dative alternation, all known errors involve the overgeneralisation of prepositional-object (PO) verbs into the double-object (DO) dative construction (e.g. *Don't say that to me* → *Don't say me that [38]). We are aware of no reported cases of DO verbs being overgeneralised into the PO construction (e.g. *Homer bet Marge $10* → *Homer bet $10 to Marge). With regard to the causative alternation, the vast majority of errors involve the overgeneralisation of intransitive-only verbs into the transitive-causative construction (e.g. *She cried* → *You cried her [39]). The converse error, whilst attested (e.g. *I didn't lose it* → *It won't lose [40]), is extremely rare. However, the locative is truly bidirectional, with many examples reported in the literature of ground-only verbs being used in the figure locative (e.g. *I'm going to cover myself with a screen* → *I'm going to cover a screen over me [6]) and of figure-only verbs being used in the ground locative construction (e.g. *I'm gonna pour water onto it* → *I'm gonna pour it with water [1]).

This bi-directionality of errors is a useful feature of the locative, because it allows us to test for a possible confound: that children may be completing the judgment task using task-based strategies, especially for novel verbs. For example, in the causative study of Ambridge et al. [22] and the dative study of Ambridge et al. [36], a task-based strategy of always rating intransitives (in the former) or prepositional-object datives (in the latter) as acceptable would yield adult-like judgments for these sentence types, since all were, in fact, grammatical. Note that, in principle, children could quite easily establish such a strategy on the basis of the high frequency, familiar verbs in the studies (e.g. *Bart laughed; Homer gave a book to Marge*), and apply this strategy to lower frequency and novel verbs.

Thus, of the three argument structure alternations studied with respect to the problem of the retreat from overgeneralisation - in/transitive, dative and locative - the latter constitutes the strongest test case for both the semantic verb class and frequency hypotheses. It is therefore perhaps surprising that, of the three alternations, the locative has received by far the least experimental attention. We are aware of only three relevant studies: Gropen, Pinker, Hollander and Goldberg [41] [42] and Ambridge, Pine and Rowland [13]. Both Gropen et al. studies showed support for Pinker's broad semantic classes, and Ambridge et al. found some support for both levels of semantic class as well as frequency. However, Ambridge et al. investigated the semantic verb class hypothesis using known locative verbs; no novel verbs were included. Although the authors controlled for attested usage by using verb frequency as a predictor in the regression analysis, for familiar verbs, the extent to which participants are basing their ratings on semantics alone, as opposed to attested usage, is difficult to ascertain.

### The present study

The aim of the present study was to conduct a particularly strong test of the semantic verb class and frequency hypotheses by (a) focussing on the locative alternation, and (b) including both familiar and novel verbs. We obtained grammaticality judgment data from children (aged 5–6 and 9–10) and adults for uses of high frequency, low frequency and novel locative verbs (figure-only, ground-only and alternating) in both locative constructions. We tested whether participants would be able to use verb semantics to determine the grammaticality of sentences containing novel verbs, as predicted by the semantic verb class hypothesis. We also tested whether participants' tolerance of overgeneralisation errors when verbs are used in the inappropriate construction decreased with each increasing level of verb frequency (novel/low/high), as predicted by the frequency hypothesis.

A noteworthy aspect of this study is the fact that participants were taught novel verbs, each of which had semantics consistent with only one of Pinker's narrow subclasses [2]: two novel verbs each from a ground-only subclass, a figure-only subclass and an alternating subclass. Participants' ability to use the semantics of each novel verb to make their grammaticality judgments is key to Pinker's proposal [2]: without having the necessary subclasses in place, participants will be unable to judge which locative construction is (un)grammatical for each novel verb.

## Method

### Ethics statement

This study was approved by the University of Liverpool Ethics Committee. Informed consent was obtained in writing both from adult participants and from the parents of the children who took part.

### Participants

The participants were 20 children aged 5–6 years (5;6–6;5. *M* = 5;11), 20 children aged 9–10 years (9;6–10;5, *M* = 9;11) and 20 adults aged 20–25 years. The children were recruited from primary schools, and the adults from the University of Liverpool. All participants were monolingual speakers of English, and had no known language impairments.

### Design and Materials

#### Design

The experiment used a 3×2×3×3×2 mixed design. The between-subjects variables were age of participant (5–6 years, 9–10 years, adult) and counterbalance version (two groups based on which novel verb forms were paired with each meaning). The within-subjects variables were semantic verb subclass (*fill*-type, *spray*-type, *pour*-type; see below), verb frequency (high, low, novel) and sentence type (ground-locative, figure-locative).

#### Test sentences and animations

Table 1 shows all verbs and test sentences used. Locative verbs were chosen based on Pinker's narrow subclasses [2] (subsequently referred to simply as ‘classes’). The first of these is the ground-only (or container-only) *fill* class in which “a layer completely covers a surface”, the second is the figure-only (or contents-only) *pour* class in which “a mass is enabled to move via the force of gravity”, and the third is the alternating *spray* class in which “force is imparted to a mass, causing ballistic motion in a specified direction along a trajectory”. For each class, two high frequency and two low frequency verbs with similar semantics were chosen. (Mean lemma frequency counts from the British National Corpus [43] are 5923 [range 750–18726] for high frequency verbs and 351 [range 111–658] for low frequency verbs; see Table 2 for details.) Participants were also taught novel verbs with similar meanings to the known verbs, two for each semantic class (see below for details of the training method). The form-meaning pairings for novel verbs differed for each counterbalance group in order to control for any effect of phonological form.

**Table 2:** All verbs and test sentences used in test trials.

Verb Class	Frequency	Verb	Sentence Type	Sentence
*Fill* verbs	High (18726)	Cover	*Figure	*Bart covered mud onto Lisa
			Ground	Bart covered Lisa with mud
	Low (487)	Coat	*Figure	*Bart coated mud onto Lisa
			Ground	Bart coated Lisa with mud
	Novel	bredge/blafe	*Figure	*Bart bredged/blafed mud onto Lisa
			Ground	Bart bredged/blafed Lisa with mud
	High (10546)	Fill	*Figure	*Lisa filled paper into the box
			Ground	Lisa filled the box with paper
	Low (111)	Line	*Figure	*Lisa lined paper into the box
			Ground	Lisa lined the box with paper
	Novel	chool/tesh	*Figure	*Lisa chooled/teshed paper into the box
			Ground	Lisa chooled/teshed the box with paper
*Spray* verbs	High (750)	Spray	Figure	Lisa sprayed water onto the roses
			Ground	Lisa sprayed the roses with water
	Low (544)	Sprinkle	Figure	Lisa sprinkled water onto the roses
			Ground	Lisa sprinkled the roses with water
	Novel	tesh/bredge	Figure	Lisa teshed/bredged water onto the roses
			Ground	Lisa teshed/bredged the roses with water
	High (750)	Splash	Figure	Homer splashed water onto Marge
			Ground	Homer splashed Marge with water
	Low (111)	Spatter	Figure	Homer spattered water onto Marge
			Ground	Homer spattered Marge with water
	Novel	dape/nace	Figure	Homer daped/naced water onto Marge
			Ground	Homer daped/naced Marge with water
*Pour* verbs	High (3461)	Pour	Figure	Homer poured water into the cup
			*Ground	*Homer poured the cup with water
	Low (658)	Drip	Figure	Homer dripped water into the cup
			*Ground	*Homer dripped the cup with water
	Novel	nace/dape	Figure	Homer naced/daped water into the cup
			*Ground	*Homer naced/daped the cup with water
	High 1306)	Spill	Figure	Marge spilt juice onto the rug
			*Ground	*Marge spilt the rug with juice
	Low (195)	Dribble	Figure	Marge dribbled juice onto the rug
			*Ground	*Marge dribbled the rug with juice
	Novel	blafe/chool	Figure	Marge blafed/chooled juice onto the rug
			*Ground	*Marge blafed/chooled the rug with juice

Verb frequency counts (lemma counts from the British National Corpus, [46]) are provided in brackets.

For each of the verbs, a test sentence was created using each of the figure- and ground-locative constructions (see Table 2). Thus, for each verb in the ground-only *fill* class and the figure-only *pour* class, one sentence for each verb was grammatical and one ungrammatical (e.g. *Lisa filled paper into the box; Lisa filled the box with paper; Homer poured water into the cup; *Homer poured the cup with water), whereas both sentences were grammatical for verbs in the alternating *spray* class (e.g. *Lisa sprayed the roses with water; Lisa sprayed water onto the roses*). Both sentences in each pair contained identical noun phrases.

For all test sentences, animations were created using Anime Studio Pro Version 5.5 [44] and presented to participants using a laptop computer. Animations for both sentences in each test pair were identical, but each was presented with the relevant pre-recorded test sentence. Animations served to ensure that participants understood the intended meaning of the sentences, particularly those including novel verbs. They also established the veracity of each of the descriptions, thereby encouraging the participants, particularly the younger ones, to judge the sentences on the basis of their grammaticality rather than their truth value.

#### Novel verb training sentences and animations

Each novel verb was assigned a meaning similar to, but subtly different from, its semantic classmates in the study, whilst still being consistent with the class (e.g. filling *with a particular substance* or pouring *in a particular manner*; see Table 2). The English language includes verbs specifying both filling/coating *with a particular substance* (e.g. *to oil, to water, to paper*) and pouring *in a particular manner* (e.g. *to dribble, to drip, to ladle*). Thus, these novel verb meanings are neither non-language-like in general nor non-English-like in particular.

For each novel verb, three animations were created in order to convey the intended meanings to participants. For each of these animations, the novel verb was given three times, always as a gerund. The sentences were as follows:

1. (before clip) Look what CHARACTER's gonna do, it's called VERBing.
2. (during clip) Look what CHARACTER's doing, it's called VERBing.
3. (after clip) So VERBing is [followed by a brief definition, see Table 3].

**Table 3:** Novel verbs and definitions.

Novel verb	Definition
Novel cover/coat	like covering, except that it has to be with mud (like this)
Novel fill/line	like filling, except that it has to be with paper (like this)
Novel spray/sprinkle	like spraying, except that you have to press a button (like this)
Novel splash/spatter	like splashing, except that it has to be in big blobs (like this)
Novel pour/drip	like pouring, except that it has to be in one big lump (like this)
Novel spill/dribble	like spilling, except that it has to be straight down in tiny drops (like this)

The definitions were intended to clarify the meanings of each verb and point out the important features of the action, which would enable learners to recognise each verb as being consistent with the intended narrow semantic class. Importantly, novel verbs were never presented in locative or transitive sentences during training (only as simple intransitives), to prevent participants basing their judgments of the novel-verb sentences on attested usage. Rather, according to the semantic verb class hypothesis, learners should determine the locative construction(s) in which each verb can be used on the basis of its semantics.

#### Grammaticality Judgments

**Figure 1:**
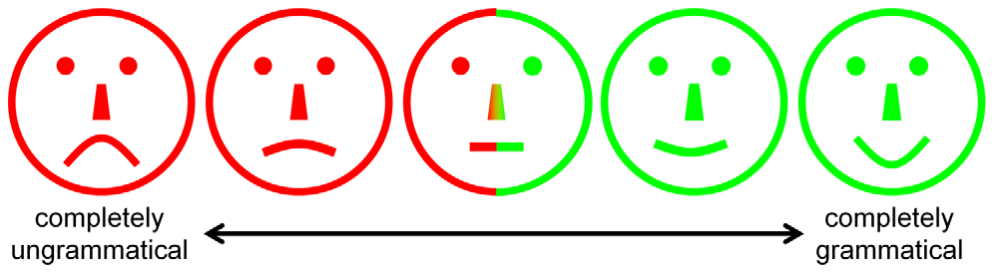
Five-point ‘smiley face’ scale for providing grammaticality judgments.

Participants rated sentences for grammatical acceptability using a five-point ‘smiley face’ scale (see Figure 1 and [22]). The scale was presented with no text or numbers. After viewing an animation and hearing the accompanying sentence, children were asked to first choose a coloured counter, with green indicating that the sentence ‘sounded good’ and red that it ‘sounded silly’. They then placed the counter onto the scale to indicate how ‘good’ or ‘silly’ it sounded. The use of counters was intended to enable younger children to indicate that they found a sentence broadly acceptable or unacceptable, even if they were unable to provide a more graded judgment (although this did not turn out to be the case). The experimenter made a note of the judgment rating the child gave for each sentence. Adults and older children were asked simply to tick one of the faces to provide their judgment rating.

Participants were trained in the use of the judgment scale with a series of seven training animations. The first four of these were designed to be clearly acceptable or unacceptable, with the others designed to receive ratings somewhere in between. Sentences were chosen based on ratings given by participants in previous studies (see Appendix S1). Ratings for the first two sentences were given by the experimenter, to demonstrate the use of the scale, and participants were given feedback on their ratings for the five subsequent sentences. No feedback was given during the experiment proper. Detailed descriptions of the training procedure are given in Ambridge et al. ([22], pages 106–107) and Ambridge ([45], pages 122–123).

### Procedure

Participants were first taught the novel verbs and then received training on the use of the grammaticality judgment scale (in both cases as described above). The main study consisted of 36 test trials: one ground-locative sentence and one figure-locative sentence using each of the six high frequency verbs, six low frequency verbs and six novel verbs (see Table 1). Sentences were presented in a pseudo-random order, such that two sentences containing the same verb were never given in succession. In order to ensure that participants remembered the intended meaning of the novel verbs, one of the training trials was repeated immediately before each test trial containing a novel verb.

## Results

Because the rating scale data are not true interval scale data, an empirical logit transformation [46] was applied (all means and SEs are reported for raw scores). All post hoc comparisons used Fisher's Least Significant Difference tests. Data are available to download from http://www.benambridge.com.

### Preliminary Analysis

A preliminary analysis, in the form of a 3×3×2 (age by verb class by sentence type) mixed ANOVA, was performed on known verbs in order to confirm that the verb type classifications (figure-only/ground-only/alternating) were correct for this group of adult participants and that children were rating the sentences as expected. Assuming that this is the case, the semantic verb class hypothesis predicts an interaction of sentence type by verb class such that ground-locative uses are preferred over figure-locative uses for verbs of the *fill* class with the reverse for verbs of the *pour* class, and no preference for the *spray* class. This analysis, and all subsequent analyses, collapsed across the two counterbalance groups (which differed only with regard to the pairings of phonological stem forms and novel verb meanings), and across the two verbs in each cell of the design.

The ANOVA yielded several main effects. However, these will not be discussed as they collapse across grammatical and ungrammatical sentences, and so are not relevant to the hypotheses of the study. Importantly, as predicted, an interaction of verb class by sentence type was observed (*F*_(2, 114)_ = 219.61, *p*<0.001, η_p_^2^ = 0.79). Analysis of this interaction revealed that, as predicted, for verbs in the *fill* class, participants significantly preferred ground-locative uses (*M* = 4.35, *SE* = 0.05) over figure-locative uses (*M* = 3.16, *SE* = 0.07, *p*<0.001). Conversely, for verbs in the *pour* class, participants significantly preferred figure-locative uses (*M* = 4.20, *SE* = 0.09) over ground-locative uses (*M* = 2.43, *SE* = 0.10, *p*<0.001). Also as expected, for verbs in the alternating *spray* class, participants showed no preference for either sentence type (ground *M* = 4.18, *SE* = 0.06; figure *M* = 4.09, *SE* = 0.07; *p* = 0.12, n.s.).

**Figure 2:**
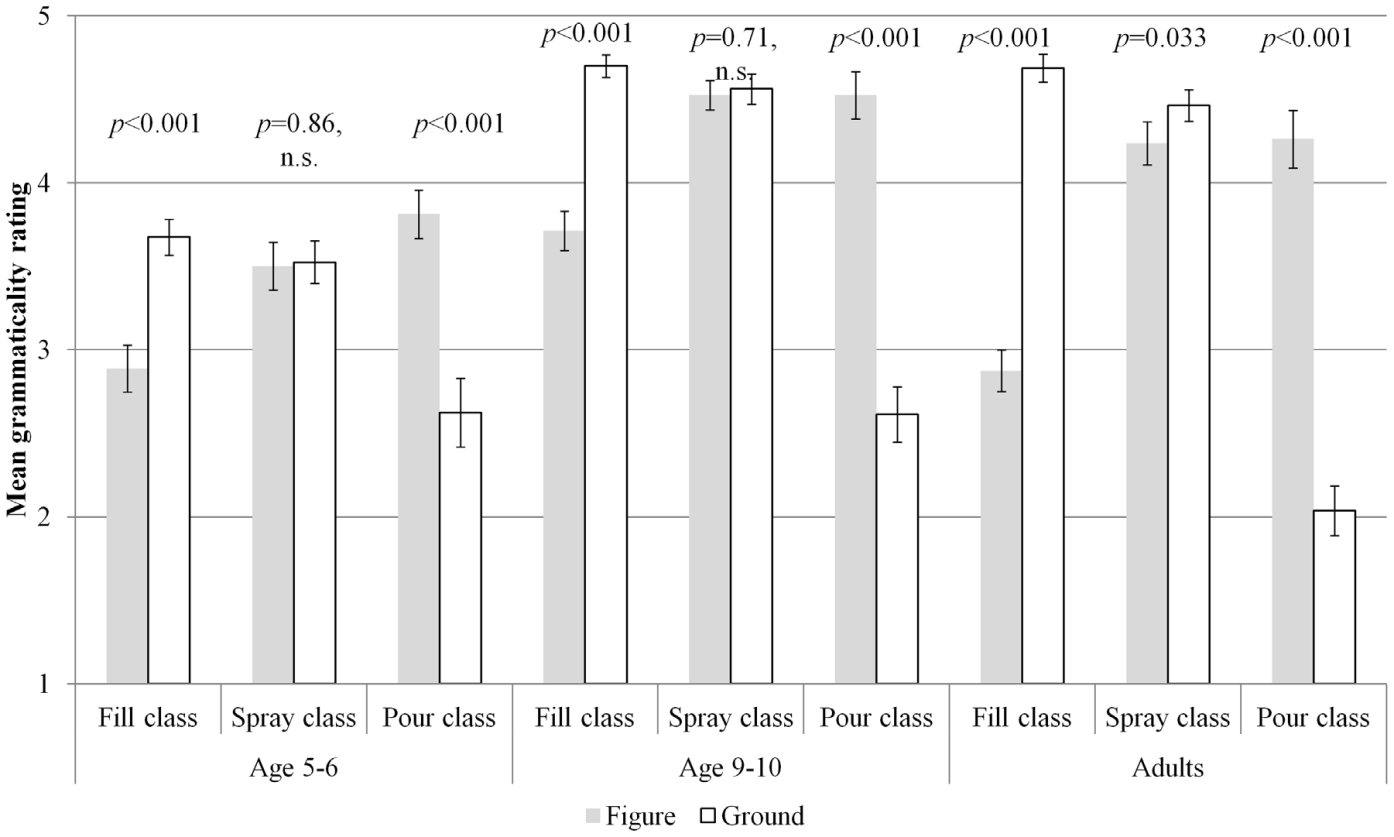
Three-way interaction of age by verb class by sentence type for familiar verbs.

A significant 3-way interaction of verb class by sentence type by age (*F*_(4,114)_ = 9.05, *p*<0.001, η_p_^2^ = 0.24) indicated that the pattern of results outlined above differed according to age group (Figure 2). This interaction was driven by the fact that, whilst all age groups displayed the predicted pattern for the non-alternating *fill* and *pour* verb classes, the adults also displayed an unexpected preference for ground-locative uses of verbs from the alternating *spray* class, although a mean rating of 4 or above still indicates that both sentence types were rated as broadly acceptable. It is possible that this result reflects adults' sensitivity to the holism constraint: when an action has been wholly and successfully completed (as is the case for the animations using alternating verbs in the present study), the ground-locative construction is more felicitous that the figure-locative construction (cf. *Lisa taught the students French* vs. *Lisa taught French to the students*). This is an issue to which we will return in the Discussion.

### Testing the semantic verb class hypothesis

In order to test the semantic verb class hypothesis, participants were taught six novel verbs, two of which were semantically consistent with the ground-only *fill* class, two with the alternating *spray* class and two with the figure-only *pour* class. Participants were then asked to judge sentences containing each of these novel verbs for their grammaticality. Each verb was presented in a figure-locative and a ground-locative construction. The semantic verb class hypothesis predicts that, as with known verbs of the same semantic classes, participants will judge figure-locative uses of the novel *fill* verbs to be less acceptable than ground-locative uses of these verbs, with the opposite pattern for the novel *pour* verbs, and no difference for the alternative uses of the novel *spray* verbs.

These predictions were again tested by means of a 3×3×2 (age by verb class by sentence type) mixed ANOVA, in this case conducted on the ratings for the novel verbs only. As before, this analysis yielded several main effects, which will not be discussed because they collapse across grammatical and ungrammatical sentences. Importantly, as predicted, and in line with the results for all verbs, an interaction of verb class by sentence type was observed (*F*_(2, 114)_ = 42.45, *p*<0.001, η_p_^2^ = 0.43). Analysis of this interaction revealed that, as predicted, for novel verbs in the ground-only *fill* class, participants significantly preferred ground-locative uses (*M* = 4.17, *SE* = 0.07) over figure-locative uses (*M* = 3.52, *SE* = 0.09, *p*<0.001). Conversely, and again as predicted, for novel verbs in the figure-only *pour* class, participants significantly preferred figure-locative uses (*M* = 4.19, *SE* = 0.08) over ground-locative uses (*M* = 3.18, *SE* = 0.13, *p*<0.001). Unexpectedly, for novel verbs in the alternating *spray* class, participants also showed a small but significant preference for ground-locative uses (*M* = 4.20, *SE* = 0.10) over figure-locative uses (*M* = 3.93, *SE* = 0.10, *p* = 0.031), although a mean rating of around 4 or above still indicates that both sentence types were rated as broadly acceptable. As previously noted, this may be due to the holism constraint.

**Figure 3:**
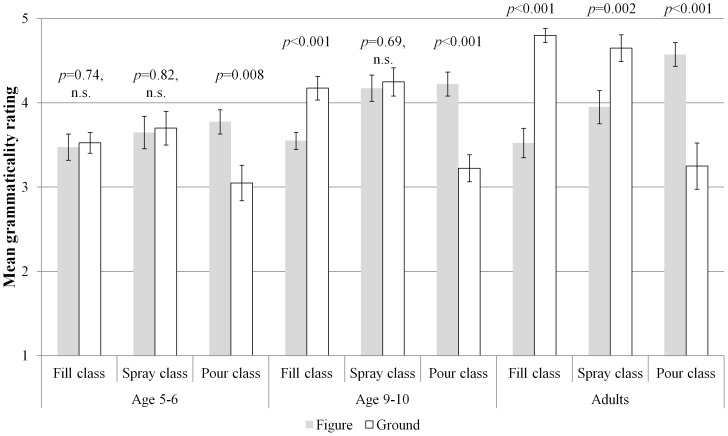
Three-way interaction of age by verb class by sentence type for novel verbs.

A significant 3-way interaction of class by sentence type by age (*F*_(4,114)_ = 4.27, *p* = 0.003, η_p_^2^ = 0.13) indicated that the pattern of results outlined above differed according to age group. As outlined in more detail below, this interaction was driven by the fact that, whilst all groups displayed the predicted pattern for the novel verbs in the non-alternating *pour* class, only older children and adults showed the expected preference for ground-locative uses of novel verbs in the non-alternating *fill* class, and only the adults displayed the unexpected preference for ground uses of novel verbs from the alternating *spray* class (see Figure 3).

As predicted by the semantic verb class hypothesis, the 5-year-olds showed no significant preference for novel alternating *spray* class verbs in figure-locative uses (*M* = 3.65, *SE* = 0.19) or ground-locative uses (*M* = 3.78, *SE* = 0.20, *p* = 0.82, n.s.). Also as predicted, they did significantly prefer figure-only *pour* verbs in figure-locative uses (*M* = 3.78, *SE* = 0.14) over ground-locative uses (*M* = 3.05, *SE* = 0.21, *p* = 0.008). These results suggest that they have identified the verb classes of these novel verbs correctly, and are using this information to judge the grammaticality of the verbs' use in different locatives. Contrary to the prediction, however, the 5-year-olds displayed no significant preference for novel ground-only *fill* class verbs in ground-locative uses (*M* = 3.53, *SE* = 0.12) over figure-locative uses (*M* = 3.48, *SE* = 0.16, *p* = 0.74, n.s.). It is possible that this youngest group of children had not fully grasped the complex semantics of *fill* class verbs, which may be more complex than those of the *pour* class (see [42] and Introduction, above).

The results for the 9-year-olds are all as predicted by the semantic verb class hypothesis: no preference for novel alternating *spray* class verbs in either figure-locative uses (*M* = 4.18, *SE* = 0.16) or ground-locative uses (*M* = 4.25, *SE* = 0.17, *p* = 0.69, n.s.), a significant preference for figure-only *pour* class verbs in figure-locative uses (*M* = 4.23, *SE* = 0.14) over ground-locative uses (*M* = 3.23, *SE* = 0.16, *p*<0.001), and a significant preference for ground-only *fill* class verbs in ground-locative uses (*M* = 4.18, *SE* = 0.14) over figure-locative uses (*M* = 3.55, *SE* = 0.10, *p*<0.001).

Adults also displayed the predicted preferences for the novel figure-only *pour* class verbs and the novel ground-only *fill* class verbs. They preferred figure-only *pour* class in figure-locative uses (*M* = 4.58, *SE* = 0.14) over ground-locative uses (*M* = 3.25, *SE* = 0.28, *p*<0.001) and they preferred novel ground-only *fill* class verbs in ground-locative uses (*M* = 4.80, *SE* = 0.08) over figure-locative uses (*M* = 3.53, *SE* = 0.18, *p*<0.001). Both of these results are in line with the predictions of the semantic verb class hypothesis. Unexpectedly, however, the adult participants also preferred the novel alternating *spray* class verbs in ground-locatives (*M* = 4.65, *SE* = 0.16) over figure-locatives (*M* = 3.95, *SE* = 0.20, *p* = 0.002). This unexpected result parallels the findings observed for adults with familiar verbs, and may again be explained by the holism constraint (see discussion). The fact that the 9-year-olds did not show this preference, whilst otherwise displaying an adult-like pattern of results, indicates that the holism constraint (as applied to the ground-locative construction) may not be fully acquired until very late in development.

### Testing the frequency hypothesis

To test the frequency hypothesis, we calculated difference scores for grammaticality judgment ratings for ‘grammatical’ sentences (ground-locative uses of *fill* class verbs; figure-locative uses of *pour* class verbs) minus ‘ungrammatical’ sentences (figure-locative uses of *fill* class verbs; ground-locative uses of *pour* class verbs) for high frequency, low frequency and novel verbs in both of these non-alternating classes. These difference scores represent the degree of preference for grammatical over ungrammatical verb uses (or perhaps more importantly for our purposes, the degree of *dispreference* for *un*grammatical verb uses relative to matched grammatical alternatives). Alternating verbs were not included in this analysis since the frequency hypothesis only makes predictions regarding the degree of unacceptability of ungrammatical verb uses (for alternating verbs, by definition, neither figure- nor ground-locative uses are ungrammatical).

The frequency hypothesis predicts that the largest difference scores will be observed for the high frequency verbs, smaller difference scores for the low frequency verbs and the smallest difference scores for the novel verbs. That is, increased exposure to a verb in grammatical sentences is predicted to increase the strength of the inference that non-attested uses are not permitted, and hence the extent to which participants will rate ungrammatical uses of that verb as unacceptable.

A 3×2×3 (age by verb class by verb frequency) ANOVA revealed that all three main effects were significant. The main effect of verb class (*F*_(1,57)_ = 29.83, *p*<0.001, η_p_^2^ = 0.34) indicates that participants showed a larger dispreference for ungrammatical uses of *pour* class verbs (*M* = 1.52, *SE* = 0.10) than *fill* class verbs (*M* = 1.01, *SE* = 0.06). While the frequency hypothesis makes no predictions about verb class, this result is consistent with the results of the semantic verb class analysis, which found that participants were less tolerant of overgeneralisation errors with novel *fill*-type verbs than novel *pour*-type verbs.

The main effect of age (*F*_(2,57)_ = 18.08, *p*<0.001, η_p_^2^ = 0.39) demonstrates that adults (*M* = 1.78, *SE* = 0.12) showed a greater degree of dispreference for ungrammatical sentences than both 9-year-olds (*M* = 1.24, *SE* = 0.08) and 5-year-olds (*M* = 0.79, *SE* = 0.14), and that 9-year-olds showed a greater degree of dispreference for such uses than 5-year-olds (all comparisons were significant at *p*<0.01 or better). This result could be interpreted as showing support for the frequency hypothesis, as adults will have had more exposure to grammatical uses of the relevant verbs than 9-year-olds who, in turn, will have had more exposure than 5-year-olds. For this interpretation to be correct, the important factor would have to be *absolute* frequency of exposure to the verbs in competing constructions (e.g. total number of ground-locative uses of *fill*), which obviously increases with age, as opposed to *relative* frequency (e.g. proportion of uses of *fill* in the ground-locative construction as opposed to other constructions), which presumably stays relatively constant across development. However, the lack of interaction between age and verb frequency (see below) suggests that this is not the case. That is, adults did not display a larger frequency effect (i.e. larger between-verb differences) than children, which one would expect if the relevant factor were absolute differences in verb frequency. It is therefore likely that the main effect of age was simply due to older participants performing better on the task.

**Figure 4:**
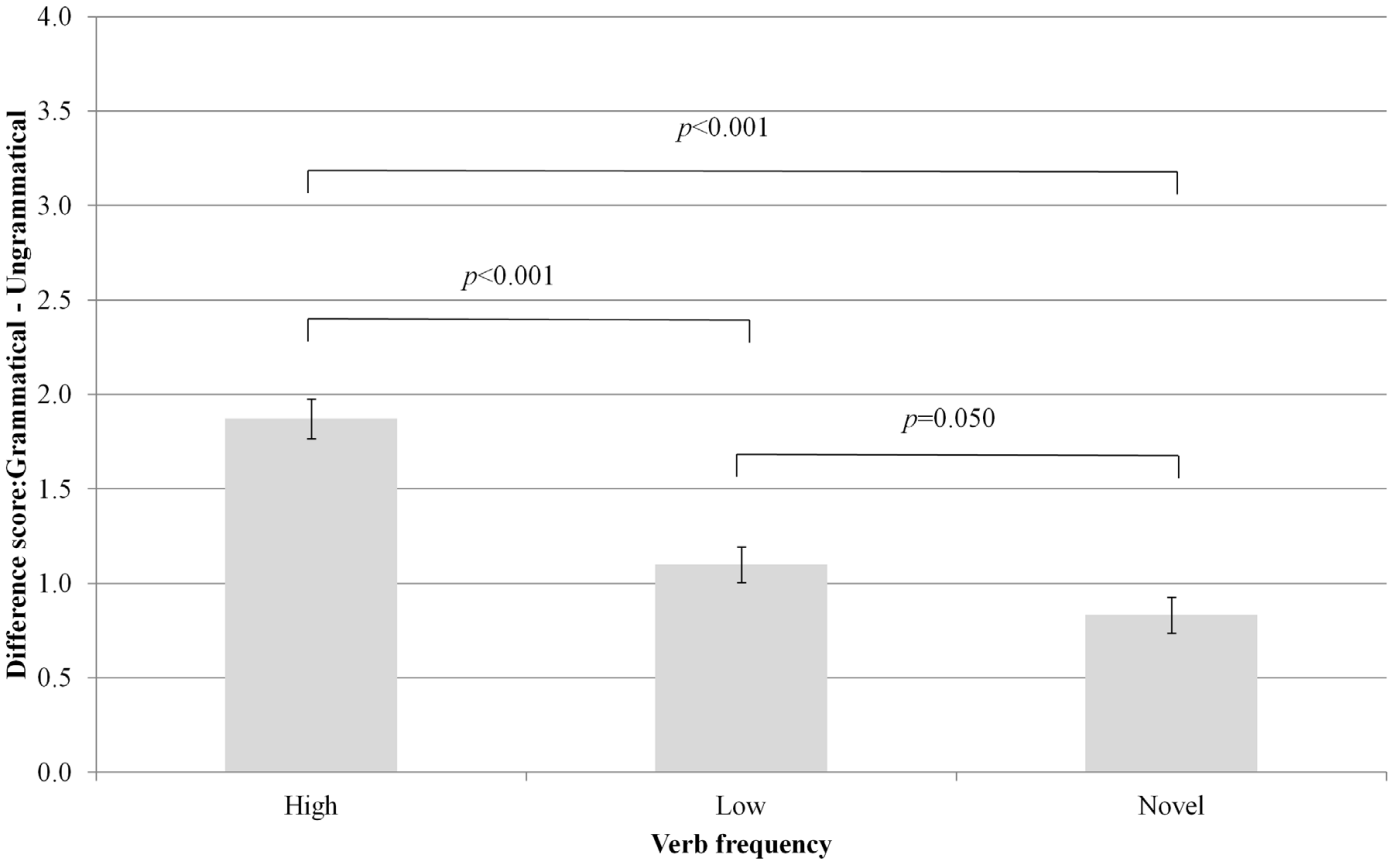
Main effect of verb frequency.

Importantly, as predicted by the frequency hypothesis, a main effect of verb frequency was observed (*F*_(2,114)_ = 38.25, *p*<0.001, η_p_^2^ = 0.40; Figure 4) such that participants showed a greater dispreference for ungrammatical uses of the high frequency verbs (*M* = 1.87, *SE* = 0.11) than either the low frequency verbs (*M* = 1.10, *SE*  = 0.09, *p*<0.001) or the novel verbs (*M* = 0.83, *SE* = 0.10, *p*<0.001), which also differed significantly from each other in the predicted direction (*p* = 0.050), although this last difference was much smaller.

The analysis revealed no significant interactions of frequency by age (*F*_(4,114)_ = 0.17, *p* = 0.96, n.s., η_p_^2^ = 0.01), verb class by age,(*F*_(2,57)_ = 1.74, *p* = 0.19, n.s., η_p_^2^ = 0.06), verb class by frequency (*F*_(2,114)_ = 1.84, *p* = 0.16, n.s., η_p_^2^ = 0.03) or frequency by verb class by age (*F*_(4,114)_ = 0.94, *p* = 0.45, n.s., η_p_^2^ = 0.03).

## Discussion

The aim of the present study was to conduct a particularly strong test of the semantic verb class hypothesis [2] and the frequency hypothesis (e.g. [11], [12]) by (a) focussing on the locative alternation, and (b) including both familiar and novel verbs. To this end, we obtained, from children (aged 5–6 and 9–10 years) and adults, judgments of figure- and ground-locative sentences containing high frequency, low frequency and novel verbs consistent with figure-only, ground-only and alternating narrow semantic classes.

The findings suggest that, in general, participants were able to use the semantics of each novel verb to align them with the ground-only *fill* class, the alternating *spray* class or the figure-only *pour* class, although the youngest group of children were unable to do so for novel *fill*-type verbs, and adults showed an unexpected preference for ground-locative uses of novel *spray*-type verbs. The findings of the present study also provide support for the frequency hypothesis: participants in all age groups displayed a greater dispreference for overgeneralisation errors with high frequency than with low frequency familiar verbs, and for errors with both of these groups than with novel verbs.

### The role of semantics

According to Pinker's semantic verb class hypothesis [2], locative verbs fall into one of two broad semantic classes. A broad-range rule links entries for alternating verbs such as *spray*, which appear in both broad classes, allowing verbs attested in one locative construction to be used in the other (e.g. *Lisa sprayed the flowers with water → Lisa sprayed water onto the flowers*). Overgeneralisation errors occur when this rule is incorrectly applied to non-alternating verbs, such as *fill* and *pour*, and cease only when children acquire the more specific narrow semantic subclasses and narrow-range rules that allow the alternation to be restricted to verbs whose semantics are compatible with the core meanings of both locative constructions.

The main test of Pinker's hypothesis in the current study involved novel verbs. Participants were taught six novel verbs with semantics consistent with one of Pinker's narrow subclasses of locative verbs: two each were consistent with (a) the ground-only *fill* class, (b) the figure-only *pour* class, and (c) the alternating *spray* class. Participants provided grammaticality judgments for ground-locative and figure-locative uses of each of the novel verbs with results showing that, as predicted, participants judged ground-locative uses of novel *fill*-type verbs to be significantly more acceptable than figure-locative uses of these verbs, with the opposite pattern observed for novel *pour*-type verbs. Since these verbs were never presented in locative constructions during training, participants must have been using verb semantics, as opposed to attested usage, to make these judgments. The subtle differences between subclasses of locative verbs, which are also not easily observable, make the locative alternation a particularly strong test of the semantic verb class hypothesis. In addition, the fact that both some figure-locative and some ground-locative sentences were ungrammatical allows us to rule out the possibility that participants were using a task-based strategy to identify the ungrammatical sentences (cf. [22], [36]). Thus, the results of this study clearly point to an important role for verb semantics in the retreat from overgeneralisation errors in the locative construction.

The semantic verb class hypothesis predicts no preference for either locative construction for alternating *spray*-type verbs. However, while both constructions were judged to be broadly grammatical, adult participants demonstrated an unexpected preference for ground-locative uses of both familiar verbs and novel verbs conforming to the semantics of this subclass. Therefore, one possibility is that adults simply have a general preference for the ground-locative construction for alternating verbs (although this is inconsistent with a strict interpretation of Pinker [[2], page 127], who lists *spray*-type verbs as being “content-oriented”, such that any preference involving these alternating verbs should have been for the figure-locative construction).

A possible explanation for the unexpected preference for ground-locative uses of alternating verbs can be found in the holism constraint. This constraint applies to ground-only locative verbs such as *fill* and *cover*, where the object must be completely filled or covered, respectively, in order for the sentence to be an accurate description of the event. The constraint also applies to the ground-locative construction itself: one semantic feature of this construction, but not the figure-locative construction, is that the ‘ground’ (e.g. the container) must be wholly affected. Indeed, it is the incompatibility of the semantics of the figure-locative construction and the semantics of verbs such as *fill* and *cover* that makes figure-locative sentences using these verbs ungrammatical.

It is possible that participants may have preferred the ground-locative uses of alternating verbs included in this study because, in the training for the novel verbs and all test animations, the ‘location’ or ‘ground’ was always completely affected (e.g. water splashed onto all of it). It was necessary to create the animations in this way in order to keep the same methodology across all verbs and classes, since, without being completely splashed with water, the animation would have been inconsistent with the ground-locative construction. The animations could therefore be considered to be more consistent with the semantics of the ground- locative construction than figure-locative construction. The results also suggest a developing knowledge of the holism requirement, as applied to individual verbs, between the age of 5 and adulthood, which in turn provides further support for the semantic verb class hypothesis. Unlike the older children and adults, the 5- to 6-year-olds preferred figure-locative uses of novel *pour*-type verbs but showed no preference for either argument structure for novel *fill*-type verbs. This suggests that these children were unable to appreciate the holism requirement of the novel *fill*-type verbs they were taught based on the animations they viewed during training (see also [42]). The disparity between young children's judgment data with novel and familiar verbs may also indicate that these children are basing their grammaticality judgments with familiar verbs on attested usage as opposed to, or in addition to, verb semantics.

Additional support for the importance of a developing knowledge of the holism constraint, as applied to the ground-locative construction, is the fact that only the adult participants gave different judgment scores for the two locative uses of alternating *spray*-type verbs (for both known and novel verbs), although both constructions were judged to be broadly grammatical. This indicates knowledge of the importance of context to the semantics of the alternative locative constructions themselves, which may not yet have developed in the children we tested, leading adults to judge ground-locative uses of *spray*-type verbs as more acceptable than figure-locative uses of these verbs, based on the animations they viewed.

### The role of frequency

The frequency hypothesis (e.g. [11], [12]) emphasises the importance of statistical properties of the input in children's language acquisition. Under this hypothesis, children retreat from overgeneralisation errors by inferring, from their absence in the input, that certain argument structures cannot be used with certain verbs. The more a child hears, for example, the verb *fill* used in different constructions with a similar meaning (preemption) or a different construction of any kind (entrenchment) without also hearing it in the figure-locative construction, the better able they are to determine that it is not possible to use *fill* in the latter. This hypothesis therefore predicts that participants will judge overgeneralisation errors with high frequency verbs to be less acceptable than equivalent overgeneralisation errors with low frequency verbs.

Results from the current study provide support for the frequency hypothesis. Participants of all ages showed the same patterns of dispreference for overgeneralisation errors, with higher dispreference scores for such errors with high frequency verbs, lower scores for low frequency verbs, and the lowest dispreference scores for novel verbs, which essentially have a frequency of zero. This finding replicates that of Ambridge et al. [13], who found a negative correlation between verb frequency and the acceptability of errors across a wider range of locative verbs. So, despite the low frequency of locative verbs and constructions in the input, the effects of this mechanism can clearly be seen in all age groups tested here.

The frequency hypothesis could be interpreted in two ways: either *absolute* frequency of a verb or the *relative* frequency of that verb in competing constructions could be taken as the important factor in the retreat from overgeneralisation. Initially, the finding that participants' dispreference for overgeneralisation errors increased with age appears to show support for the interpretation favouring absolute frequency, since the absolute frequencies of the relevant verbs in different constructions will increase with age, whilst the relative frequencies are likely to remain fairly constant throughout development. However, the fact that no interaction between age and verb frequency was observed counts against this interpretation. Provided that the ratio of high to low frequency verbs in the input remains relatively stable for all ages, an absolute frequency interpretation of the frequency hypothesis would have predicted an increasing difference in dispreference scores for overgeneralisation errors between verbs of different frequencies as the age of participants increased. The main effect of age observed here is therefore likely to be due to older participants simply performing better on the task. So, whilst the present study did not specifically investigate this aspect of the frequency hypothesis, findings suggest that the relative frequency of a verb in competing constructions might be the most important statistical factor in the retreat from overgeneralisation.

### Explaining the retreat from overgeneralisation

The predictions of both the semantic verb class hypothesis and the frequency hypothesis have been supported by the findings of the current study: semantics and statistics clearly both have a role to play in the retreat from overgeneralisation. However, neither of these accounts in its current form can explain both the frequency effect and the fact that participants were able to provide grammaticality judgments for novel verbs in line with those of semantically-related familiar verbs. In order to explain the retreat from overgeneralisation errors more fully, an account must be posited that can explain both of these effects, such as Perfors et al.'s Bayesian account [28] or Ambridge et al.'s FIT account [13] [47] (see also [48]–[51]).

This study has shown that, as predicted by the semantic verb class hypothesis, children and adults are able to use the semantics of novel verbs to judge their grammaticality in locative sentences in line with verbs with similar semantics. As predicted by statistical learning accounts, children and adults judge errors with high frequency verbs to be worse (in comparison with their grammatical counterparts) than errors with low frequency verbs, which in turn are judged to be worse than errors with novel verbs. Thus, this paper adds to previous research indicating the importance of both semantics and statistics in children's retreat from overgeneralisation errors, and in language acquisition more widely. Future empirical and computational work should focus on testing accounts, such as those mentioned here, that integrate both of these mechanisms.

## Supporting Information

Appendix S1 Grammaticality judgment training sentences. ‘Sentences’ used in the grammaticality judgment training trials, with their ‘typical’ scores (based on Ambridge et al., 2008). The experimenter completed the first two trials to demonstrate, with participants completing the remainder. Feedback was provided if judgments were thought to be inappropriate.
(DOCX)
