# Supplementary material for: The Retreat from Locative Overgeneralisation Errors: A Novel Verb Grammaticality Judgment Study
Source: PLoS One. 2014 May 15;9(5):e97634. doi: 10.1371/journal.pone.0097634 (PMC4022747; doi:10.1371/journal.pone.0097634)
Supplement: Appendix S1 — Grammaticality judgment training sentences. ‘Sentences’ used in the grammaticality judgment training trials, with their ‘typical’ scores (based on Ambridge et al., 2008). The experimenter completed the first two trials to demonstrate, with participants completing the remainder. Feedback was provided if judgments were thought to be inappropriate. (DOCX) [file pone.0097634.s001.docx]

**Appendix S1. Grammaticality judgment training sentences.** ‘Sentences’ used in the grammaticality judgment training trials, with their ‘typical’ scores (based on Ambridge et al., 2008). The experimenter completed the first two trials to demonstrate, with participants completing the remainder. Feedback was provided if judgments were thought to be inappropriate.

| Sentence | Typical score |
| --- | --- |
| The frog caught the fly | 5 |
| His teeth man the brushed | 1 |
| The cat drank the milk | 5 |
| The dog the ball played with | 1 |
| The man tumbled Bart into a hole | 2 or 3 |
| The magician vanished Bart | 2 or 3 |
| The funny clown giggled Bart | 1 or 2 |
